# Programmatic Use of Molecular Xenomonitoring at the Level of Evaluation Units to Assess Persistence of Lymphatic Filariasis in Sri Lanka

**DOI:** 10.1371/journal.pntd.0004722

**Published:** 2016-05-19

**Authors:** Ramakrishna U. Rao, Sandhya D. Samarasekera, Kumara C. Nagodavithana, Manjula W. Punchihewa, Tharanga D. M. Dassanayaka, Gamini P. K. D, Ethan Ford, Udaya S. B. Ranasinghe, Ralph H. Henderson, Gary J. Weil

**Affiliations:** 1 Department of Internal Medicine, Infectious Diseases Division, Washington University School of Medicine, St. Louis, Missouri, United States of America; 2 Anti Filariasis Campaign, Sri Lanka Ministry of Health, Colombo, Sri Lanka; 3 Regional Anti Filariasis Unit, Galle, Sri Lanka; 4 Task Force for Global Heath and NTD Support Center, Atlanta, Georgia, United States of America; University of South Florida, UNITED STATES

## Abstract

**Background:**

Sri Lanka’s Anti Filariasis Campaign distributed 5 rounds of mass drug administration (MDA with DEC plus albendazole) to all endemic regions in the country from 2002–2006. Post-MDA surveillance results have generally been encouraging. However, recent studies have documented low level persistence of *Wuchereria bancrofti* in Galle district based on comprehensive surveys that include molecular xenomonitoring (MX, detection of filarial DNA in mosquitoes) results. The purposes of this study were to demonstrate the use of MX in large evaluation units (EUs) and to field test different mosquito sampling schemes.

**Methodology/Principal Findings:**

Galle district (population 1.1 million) was divided into two EUs. These included a coastal EU with known persistent LF and an inland EU with little persistent LF. Mosquitoes were systematically sampled from ~300 trap locations in 30 randomly selected clusters (health administrative units) per EU. Approximately 28,000 *Culex quinquefasciatus* were collected with gravid traps and tested for filarial DNA by qPCR. 92/625 pools (14.7%) from the coastal EU and 8/583 pools (1.4%) from the inland EU were positive for filarial DNA. Maximum likelihood estimates (MLE) for filarial DNA rates were essentially the same when the same number of mosquito pools were collected and tested from 75, 150, or 300 trap sites (range 0.61–0.78% for the coastal EU and 0.04–0.07% for the inland EU). The ability to use a smaller number of trap sites reduces the cost and time required for mosquito sampling.

**Conclusions/Significance:**

These results suggest there is widespread persistence of *W*. *bancrofti* infection in the coastal Galle EU 8 years after the last round of MDA in 2006, and this is consistent with other data from the district. This study has shown that MX can be used by national programs to assess and map the persistence of *W*. *bancrofti* at the level of large EUs in areas with *Culex* transmission.

## Author Summary

Lymphatic filariasis (LF) is a disabling neglected tropical disease that affects millions of people in 73 countries. The Global Program to Eliminate Lymphatic Filariasis (GPELF) is using mass drug administration (MDA) to reduce infections in humans below levels required for sustained transmission by mosquitoes. Prior studies have shown that molecular xenomonitoring (MX, detection of filarial DNA in insect vectors) is useful for detecting low-level persistence of *W*. *bancrofti* infection following MDA, but technical challenges have prevented national programs from adopting this approach on a large scale. This paper reports the first use of MX for post-MDA surveillance in large evaluation units. CDC gravid traps were effective for collecting large numbers of *Culex quinquefasciatus* mosquitoes, and filarial DNA was detected by qPCR. We found that MX is more sensitive than other surveillance methods for detecting persistent *W*. *bancrofti* in communities and that there is widespread, low-level persistence of infection in coastal Galle district (Sri Lanka) 8 years after the last round of MDA. The systematic mosquito sampling protocol used in this study should be feasible for use by national filariasis elimination programs to assess and map the persistence of *W*. *bancrofti* in regions where the infection is transmitted by *Culex* mosquitoes.

## Introduction

Lymphatic filariasis (LF) is a disfiguring and disabling disease that affects approximately 120 million people in 73 countries. The World Health Organization (WHO) initiated a Global Programme to Eliminate Lymphatic Filariasis (GPELF) in 2000 that is largely based on repeated annual cycles of mass drug administration (MDA) with albendazole together with either ivermectin (in Africa) or diethylcarbamazine (outside of Africa) to reduce infection rates and interrupt transmission. GPELF aims to eliminate LF as a public health problem in all countries by the year 2020. With a target population of 1.4 billion, this is easily the largest disease intervention program initiated to date based on MDA; more than 6 billion medication doses were distributed to some 600 million people between 2000 and 2014 [1].

MDA has dramatically reduced LF infection rates and prevented new cases in many countries [2,3]. However, more work is needed to develop and test methods for determining whether *W*. *bancrofti* has been eliminated from countries and regions following MDA. The WHO now recommends use of transmission assessment surveys (TAS) to demonstrate interruption of transmission. TAS surveys use systematic sampling protocol to test 1^st^ and 2^nd^ grade primary school children for filarial antigenemia, and they are powered to provide 95% certainty that infection rates in children are less than 2%. While this is a useful surveillance tool, a recent study showed that antibody testing of sentinel populations and molecular xenomonitoring (MX, detection of filarial DNA in vector mosquitoes by PCR) were more sensitive than TAS for detecting persistent *W*. *bancrofti* in Sri Lanka [4]. MX relies on the ability of mosquitoes to efficiently take up filarial parasites from human blood, and parasite DNA is detected in mosquito pools by PCR. Although MX has been used extensively in recent years to demonstrate the impact of MDA on *W*. *bancrofti* in communities [4–8], no national LF elimination program has adopted MX as a routine method for post-MDA surveillance.

Sri Lanka was one of the first countries to implement a LF elimination program based on WHO guidelines. The Sri Lankan Ministry of Health’s Anti Filariasis Campaign (AFC) distributed 5 annual rounds of MDA with albendazole plus diethylcarbamazine to all endemic regions of the country between 2002 and 2006 [9]. Various types of surveillance have been conducted since the MDA program ended in 2006. Post-MDA surveillance results (based on detection of parasites in human blood by microscopy) have consistently shown microfilaremia rates lower than the target value of 1% in all sentinel and spot check sites. However, recent studies have provided evidence of persistent LF infection and transmission in several districts, and this is especially worrisome in the Southern district of Galle.

Galle District, with a population of approximately 1.06 million, is located about 125 km south of the country’s capital in Colombo. The district area is about 1,652 km2 (636 square miles) with 73 km of coastline. Ecological conditions (annual rainfall 200–250 cm, mean temperature 25–28 C, and relative humidity levels often >80%) are favorable for mosquitoes. Survey data before and after the MDA program have consistently shown higher LF rates in coastal areas that have low-lying low plains, low hills, extensive surface water, and higher human population densities than in inland areas of the district that have steep hills and better drainage.

It is well documented that *Culex quinquefasciatus* is the major *W*. *bancrofti* transmission vector in Sri Lanka [10–13], and the traditional filariasis belt extends along the coast from Puttalam in the West around to Matara in the South. Some of these areas have poorly drained polluted water, latrine catch pits, coconut husk pits, and rice fields that are primary breeding areas for *Culex* and other types of mosquitoes [14]. A devastating Tsunami in 2004 in this coastal belt affected the topography in some areas in ways that may have increased mosquito densities and disease transmission. However, there are no mapping data available on distribution of breeding sites for *C*. *quinquefasciatus* in Sri Lanka or in Galle district.

The central Ministry of Health and Nutrition (MOH) divides districts into progressively smaller health administrative units. Galle is divided into large Ministry of Health areas (MOOH) with populations in the range of 22,000–95,000. Each MOOH area is comprised of Public Health Inspector (PHI) areas, and these sub-district health administration units are comprised of smaller Public Health Midwife (PHM) areas.

Galle had high rates of LF prior to the national LF elimination program, and government surveys have shown that sentinel sites in this district have consistently had higher microfilaria (Mf) rates than most sites in other districts in the years since the MDA program ended. Although the AFC used the district as an implementation unit for MDA, Galle was divided into two evaluation units (a high-risk coastal EU and low-risk inland EU) for monitoring and evaluation.

The AFC conducted transmission assessment surveys (TAS) in these EUs in 2012–2013 according to WHO guidelines, and both EUs easily passed the WHO threshold. However, recent surveys in two public health inspector (PHI) areas in Galle district showed high rates of filarial DNA in *Culex quinquefasciatus* and high rates of anti-filarial antibodies in primary school children [4]. These results suggested that there are hotspots with persistent *W*. *bancrofti* transmission in Galle.

The current study was conducted to test a scheme for systematic sampling of mosquitoes so that MX could complement TAS for assessing residual filariasis activity by national filariasis elimination programs. The study also aimed to map areas with persistent *W*. *bancrofti* in Galle district and to compare MX results with results from an extensive population survey for microfilaremia that was conducted by the AFC in 2013. While MX has been previously used to assess the impact of MDA on persistent *W*. *bancrofti* in populations, this study reports the first time that it has been used by a national LF elimination program to detect persistent *W*. *bancrofti* at the scale of large EUs.

## Materials and Methods

### Ethics statement

We obtained consent from household members to place CDC gravid traps on their property. The Microfilaria surveys were conducted as a public health activity by the Anti Filariasis Campaign, Sri Lanka Ministry of Health. Written consent was obtained from all adults. Participation of children required written consent from at least one parent or guardian plus assent by the child. Unique identifiers of human participants were not used in this study.

### Study location

Galle district is divided into 19 Medical Officers of Health (MOOH) divisions, and each of these are comprised of smaller health administrative units called Public Health Midwife (PHM) areas that were used as evaluation areas (EAs) for the cluster surveys described in this paper. There are 340 PHM areas in Galle district with a mean population of approximately 3,000 (range 669–8025). The district was divided into two evaluation units (EU) for post-MDA surveillance. These included a high risk coastal EU with 210 PHMs in 11 MOOH areas and a low risk inland EU with 126 PHMs in 8 MOOH areas. Three of the 11 high risk MOOH areas are not located near the coast, but in this study they were considered to be part of the high risk coastal EU based on historical LF data, detection of Mf positive cases in routine surveys, and because of they are bordering areas with high Mf rates. In the 2013 census, approximately 24,600 households were listed in the coastal EU and 17,400 households were in the inland EU.

### Mosquito sample size and sampling method

The mosquito surveys used cluster sampling based on systematic selection of households (HH) to assess prevalence of *Wuchereria bancrofti* DNA in *C*. *quinquefasciatus* species [15]. The study was designed to collect and test 300 pools of 25 mosquitoes per pool collected from 300, 150, and 75 HH locations. PHM maps, census data and voter registries were used to identify the HH locations in each of these areas. Thirty PHMs were randomly selected as evaluation areas (EAs) in each of the two EUs studied using Survey Sample Builder (SSB) software (http://www.ntdsupport.org/resources/transmission-assessment-survey-sample-builder). The sampling interval was calculated by dividing the estimated number of HH in each EU by the number of HH that were needed for mosquito trap placement in the EU (300). The starting HH for each PHM was selected at random from the census list, and other HH for trap placement in that PHM were selected using the sampling interval. Subsets of 150 and 75 trapping sites were randomly chosen from the pool of 300 per EU for collection of 2 and 4 mosquito pools per trap. To allow for anticipated 10% HH refusal rate for permitting mosquito trap placement, trap locations were increased to 320, 156, 76 or 81, respectively.

### Mosquito collection and DNA preparation

The mosquito surveys were performed between December 2013 and September 2014. The long duration was due to administrative issues and not because of time requirements for the work. Mosquitoes were collected with CDC gravid traps (Model 1712, John W. Hock Company, Gainesville, FL) with liquid bait that attracts *Cx*. *quinquefasciatus* [4,16]. The traps were placed outdoors in shaded areas adjacent to houses after obtaining consent from the residents. Traps were set to collect mosquitoes from dusk to dawn for 1 to 3 nights. A second trap was placed next to selected houses in some locations in order to collect the required number of mosquitoes. This was commonly done when four pools were required from a trapping location. Traps that did not yield enough mosquitoes to form pools were moved to a new location. Mosquitoes were collected from an average of ten (range 6–19) locations in each of the 60 PHM areas.

*Culex quinquefasciatus* were manually sorted to select only gravid, semigravid or blood fed females and pooled in groups of up to 25. Pools were placed in 2 ml seal-rite tubes and dried as previously described [4]. Tubes with mosquitoes were labeled with barcode stickers and these numbers were scanned into cellphones and associated with GPS coordinates. The dried mosquitoes were transferred to the AFC central laboratory in Colombo for DNA isolation and qPCR testing.

### DNA isolation from mosquito pools

Genomic DNA extraction of pools of mosquitoes (1–25) was performed as previously described [17]. The extracted DNA samples in 200 μl of Diethylpyrocarbonate (DEPC) treated water was stored at -20 C in 1.5 ml sterile polypropylene tubes with barcode stickers.

### PCR testing of mosquito pools for detecting *W*. *bancrofti* DNA

*W*. *bancrofti* DNA was detected in mosquito DNA samples that were tested in duplicate by quantitative PCR (qPCR) in microtiter plates with an Applied Biosystems 7300 Real Time PCR System (Life technologies, California, USA) as previously described [18,19]. Positive and negative control samples were included in each qPCR assay. Mosquito DNA samples with borderline Ct values (≥38) and those with inconsistent results in duplicate wells were retested. Samples with Ct values >38 were considered to be negative. All DNA extractions and qPCR assays were performed in AFC laboratories in Colombo.

### Community surveys for microfilaremia

The AFC conducted a large scale night blood smear survey between March and August, 2013. Households were selected for the survey using the WHO guidelines [20] based on the probability proportional to estimated size (PPES) of the population with the goal of testing approximately 3% of the population (38,000 people drawn from all PHMs in the district). A maximum of 4 people per household were sampled between the ages of 2 and 70 years. Two to four teams collected blood in night blood surveys that started no earlier than 20:30 hr. Each team included a public health inspector, a blood collector, a helper, and a supervisor. It took six months to complete the night blood surveys, but additional time was required for processing of blood smears and microscopy.

### Blood tests for *W*. *bancrofti* microfilariae

Microfilaria (Mf) testing was performed with a measured volume of 60 μl of night blood. Finger prick blood samples were collected with One Touch Ultra Soft lancet holders with sterile, single use lancets (LifeScan, Inc., Milpitas, CA). Two-spot blood smears of 30 μl each were stained with Giemsa, and Mf were detected by microscopy.

### Data collection and data management

PHM numbers, household locations, trap numbers, pool numbers, and pool PCR test results were entered into Motorola (Motorola Solutions, Inc., Schaumburg, IL) Blur phones using preloaded survey forms with a LINKS data collection platform (http://www.linkssystem.org/). Specimens and laboratory qPCR test results were linked to trap locations using barcode stickers (Partnered Print Solutions, Dacula, GA). Data were downloaded as Microsoft Excel files (Microsoft Corporation, Redmond, WA) for analysis at AFC and at Washington University. Demographic information and Mf smear results were entered onto paper forms for the Mf surveys, and this information was later transferred into Excel spreadsheets (Microsoft Corporation, Redmond, WA).

### Spatial analysis

GPS coordinates for mosquito sampling sites were obtained with cellphones. Coordinates were plotted using ArcGIS 10.2.1 (ESRI, Redlands, CA).

### Statistical methods

qPCR results were expressed as the percentages of positive pools and trap sites by PHM and EU. Filarial DNA rates in mosquitoes (maximum likelihood estimates with 95% confidence intervals) were calculated with PoolScreen 2.0.3 software as previously described [21,22]. Separate PoolScreen estimates (maximum likelihood estimates or MLE with 95% confidence intervals) were calculated for approximately 300 pools from 300 trap sites, 300 pools from 150 trap sites, and 300 pools from 75 trap sites from 30 PHMs (evaluation areas) in each of the two EUs.

In order to assess filarial DNA rates in mosquitoes collected in Galle district as a single EU, the merged results from 2 EUs were analyzed by calculating mosquito DNA rates in 30 PHMs randomly selected from the 60 PHMs in two EUs that were sampled in the study. This process was repeated 30 times to assess variability in estimates obtained with different PHM samples. Results were analyzed by ANOVA and the significance of differences was assessed by the Tukey method. Data analysis was performed with Statistical Analysis Software (SAS, version 9.2, SAS Institute Inc. Cary, North Carolina). Some figures were produced with Graphpad Prism 6 (GraphPad Software, Inc., La Jolla, CA).

### Consent procedures

We obtained consent from household members to place CDC gravid traps on their property. The Mf surveys were conducted as a public health activity by the Anti Filariasis Campaign, Sri Lanka Ministry of Health. Written consent was obtained from all adults. Participation of children required written consent from at least one parent or guardian plus assent by the child. Unique identifiers of human participants were not used in this study. However, AFC followed up to treat Mf carriers identified during night blood surveys with anti-filarial medications according to WHO guidelines.

## Results

### Mosquito collections and filarial DNA rates in mosquitoes

Approximately 28,700 blood fed, gravid or semigravid *C*. *quinquefasciatus* mosquitoes were collected from more than 600 trap locations in 60 PHMs (30 in each EU) between December 2013 and September 2014 (Table 1). Mosquitoes were sorted into 1208 pools with 1–25 mosquitoes per pool (see Tables 1 and 2). Each pool contained mosquitoes from a single trap location. While 89.5% of pools contained 25 mosquitoes (1081/1208), some pools were smaller, because few mosquitoes were trapped in some collection sites despite placement of traps for up to 3 nights. Indeed, zero mosquitoes were collected in 45 trap locations. Factors such as higher altitude, very effective water drainage, and paradoxically, excessive rain affected collections at these locations.

**Table 1:** Filarial DNA rates in *Culex quinquefasciatus* in 2 evaluation units (EU) in Galle district, Sri Lanka.

Galle EUs	No. of MOOH areas	Human population	No. of PHMs surveyed	No. of pools tested	No. of mosquitoes tested	No. of Positive pools(%)	Filarial DNA rates %, (95% CI)
**Coastal/ high risk**	11	609,932	30	625	15,245	92(14.7)	0.63 (0.5–0.8)
**Inland/ low risk**	8	457,006	30	583	13,472	8 (1.4)	0.06 (0.02–0.11)
**Total**	19	1,066,938	60	1208	28,717	100(8.3)	0.36 (0.29–0.45)

**Table 2:** Estimated filarial DNA rates in *Culex quinquefasciatus* pools in 2 evaluation units (EU) in Galle, Sri Lanka.

EU	PHMs	Trap Locations	Samples	No. of pools	No. of mosquitoes	No. (%) of positive pools	Filarial DNA rates (%) in mosquitoes (95% CI)	No. (%) of positive mosquito trap locations	
**Coastal**	30	317	1- Pool	317	7801	44 (13.9)	0.61 (0.42–0.83)		44/317 (14)
	30	150	2- Pool^a^	300	7428	47 (15.7)	0.67 (0.47–0.91)		40/150 (27)
	30	150	2- Pool^a^	300	7341	52 (17.3)	0.78 (0.56–1.04)		43/150 (28)
** **	30	74	4- Pool	292	7163	43 (14.8)	0.65 (0.45–0.90)		26/74 (35)
**Inland**	30	317	1- Pool	317	7449	3 (0.95)	0.04 (0.01–0.12)		3/317 (1)
	30	150	2- Pool^a^	300	7119	4 (1.3)	0.06 (0.01–0.14)		4/150 (3)
	30	150	2- Pool^a^	300	6845	5 (1.2)	0.07 (0.02–0.17)		5/150 (3)
** **	30	77	4- Pool	266	6211	3 (1.1)	0.05 (0.01–0.18)		3/77 (4)

Filarial DNA rates were detected by qPCR. Rates of filarial DNA in mosquitoes (maximum likelihood and 95% CI) were estimated using PoolScreen 2.03. Filarial DNA rates in mosquitoes were very similar whether 300 pools were sampled at 300, 150, or 75 collection sites.

^a^Two sets of 150 areas with 300 pools each were randomly chosen for calculations.

**Fig 1:**
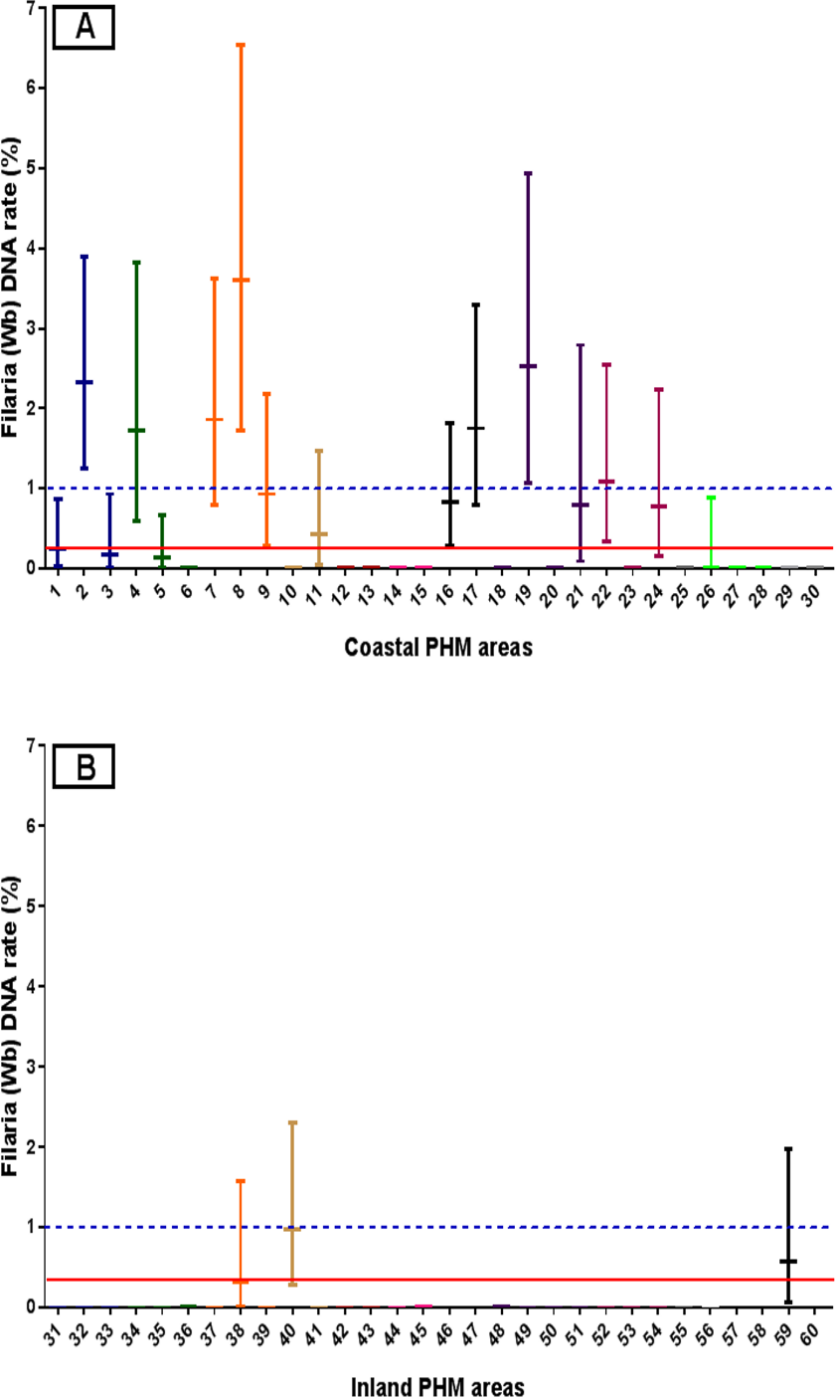
**Filarial DNA rates (MLE with 95% confidence intervals) for mosquitoes collected in the coastal (panel A) and inland (panel B) EUs in Galle district.** Lines with the same color show results from PHMs within a single MOOH area. Filarial DNA rates exceeded the target rate of 0.25% in 16 of 60 PHMs, and the upper confidence limit exceeded the target of 1% in all these 16 PHMs. The solid line in the panel A and B show the provisional target for MX. The dotted lines in the two panels are at the recommended upper confidence limits for filarial DNA rates in mosquitoes.

qPCR results by EU are summarized in Table 1. Combined results from both EUs showed that 100 of 1208 pools (8%) were positive for filarial DNA. 16/30 PHM areas in the coastal EU and 3/30 (10%) in the inland EU had at least one positive pool (range 1–16). Filarial DNA rates in mosquitoes exceeded provisional targets for filarial DNA [4,19] of 0.25% for maximum likelihood and 1% for the upper confidence limit of the estimate in 12 of 30 PHMs in the coastal EU and in 4 of 30 PHMs in the inland EU (Fig 1). Thus *W*. *bancrofti* DNA rates were high in most areas within the coastal EU and much lower in the inland EU. Some of the inland PHMs with positive qPCR results were adjacent to areas in the coastal EU with high filarial DNA rates in mosquitoes.

**Fig 2:**
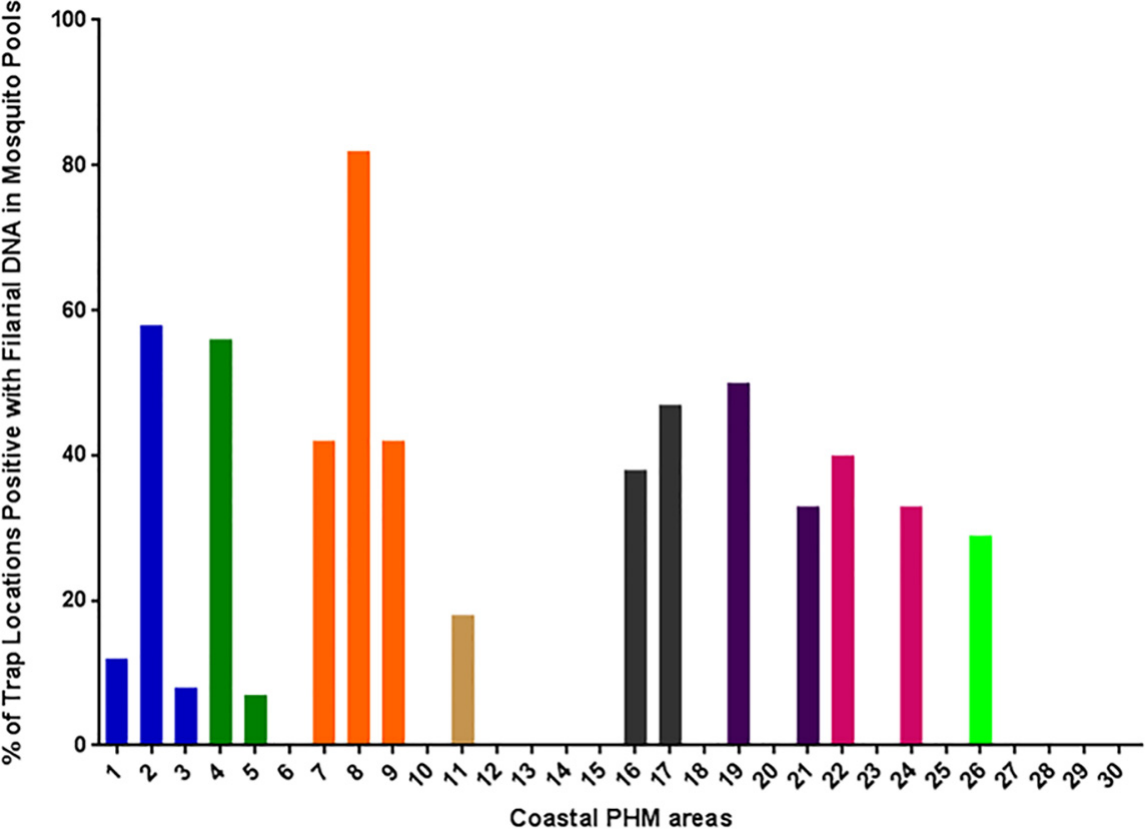
Percentages of mosquito trap locations in coastal PHM areas that yielded mosquito pools that were positive for filarial DNA by qPCR. Bars with the same color show results from PHMs within a single MOOH area. Twenty-two % of trap locations captured mosquitoes with filarial DNA, but some locations had much higher rates.

**Fig 3:**
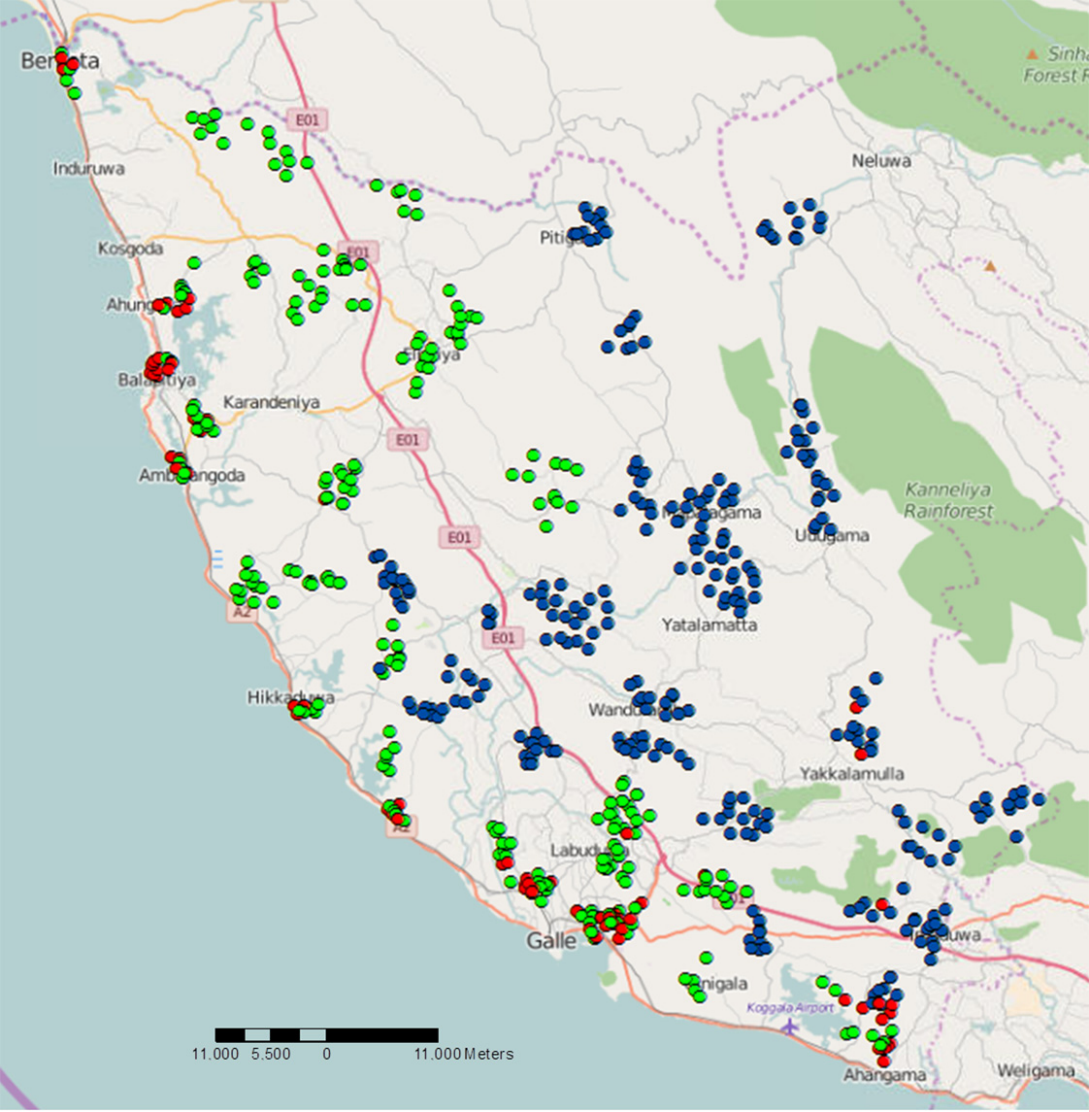
Distribution of mosquito trapping locations tested for filarial DNA in 60 PHM areas in Galle district. Molecular xenomonitoring results show trap locations with no mosquito pools positive for filarial DNA (coastal: green and inland: blue waypoints), and traps with one or more positive pools for filarial DNA are shown in red (in the coastal and inland EU areas).

Figs 2 and 3 provide information on the general location of mosquitoes with filarial DNA in the PHM areas sampled in this study. Filarial DNA was detected in mosquitoes in 22% (70/317) of trap locations in the coastal EU and in 3% (8/317) of trap locations in the inland EU. Although the number of mosquitoes collected from each trap location varied because of the sampling scheme, Fig 3 shows that mosquitoes with filarial DNA were widely distributed in coastal areas.

### Comparison of different sampling schemes for estimating rates of filarial DNA in mosquitoes

Table 2 shows that similar results were obtained when similar numbers of mosquitoes were tested in 300 pools that were collected from approximately 75, 150, or 300 sampling locations with 4, 2 or 1 pool per site, respectively. Mosquito DNA rates were also similar when different numbers of collection sites were used in the inland EU, although it was not possible to obtain enough mosquitoes for 4 pools from some collection sites.

Table 3 shows results that would have been obtained if Galle district had been considered as a single EU rather than as two EUs. The results shown are the mean and SD of 30 separate randomly selected samples of 30 EAs drawn from the entire district. The results show that the estimates were essentially the same whether mosquitoes were sampled from 316, 153, or 70 trap locations. More importantly, the results show that the parasite DNA rate in the combined EU was lower and intermediate between the rates observed in the coastal and inland EUs. Although the filarial DNA rate in mosquitoes from the district wide EU exceeded the provisional target of 0.25%, the diluting effect of results from the inland areas is clear (the MLE for the coastal EU and for the entire district were 0.67% vs. 0.35%, respectively). This emphasizes the importance of creating EU’s so that transmission risks within them are as uniform as practicable [23].

**Table 3:** Analysis of molecular xenomonitoring results using pools of *Culex quinquefasciatus* in Galle Public Health Midwife (PHM) areas.

No. of trap sites per EU	Pools per trap site	Mean No. of pools	No. of filarial DNA positive pools	MLE mean % (±SD)	*P* value^a^
316	1	316	24	0.34% (0.10)	0.9651
153	2	306	25	0.37% (0.09)	0.9867
70	4	280	23	0.35% (0.13)	0.9416

Analysis of 30 randomly selected PHMs from a total of 60 PHMs from 2 EU (mean values of 30 repetitions).

PHM areas are evaluation areas (EA)

MLE, maximum likelihood estimates using PoolScreen 2.03.

^a^*P* values are based on Chi-square for the comparison of results with different trapping protocols.

High *P* values indicate no significant difference between the means of each iteration.

### Microfilaria survey results

Night blood testing was performed with 38,065 samples collected from all PHMs and all MOOH areas in Galle district. Only 52 slides were positive for an overall rate of 0.14% (Table 4). 12 out of 19 MOOH areas recorded at least 1 Mf-positive subject (range 1–22), and the mean (SD) Mf count in positive slides was (17 ± 30; range 1–177). As expected, Mf rates were much higher in MOOH areas in the coastal EU than in the inland EU, and 47 of 52 (90.4%) Mf positives identified in the district-wide survey lived in coastal areas. The Balapitiya MOOH area in the coastal EU had the highest number of Mf-positive subjects and the highest Mf rate (0.9%); 22 of 52 (42%) Mf positive subjects were from this MOOH area. However, Mf rates were much higher (range ≥1%-4.64%) in 12 of the 340 PHMs surveyed. These were Talpe (1.39%), Panagamuwa (1.72%), Paragahathota (4.64%), Galmangoda (3.45%), Balapitiya (4.42%), Randombe (1.73%), Wellabada (1.12%), Dalawella (1.01%), Maliduwua (1.41%), Kumbhalwella-2 (2.75%), Aluthwatta (1.26%), and Mawita (1.13%), respectively. Ten of the PHMs with Mf rates >1% were in the coastal EU and two were in the inland EU.

**Table 4:** Human infection rates and parasite DNA rates from 19 MOOH areas in Galle, Sri Lanka.

		Community microfilaria (Mf) rates			Mosquito filarial DNA rates		
Evaluation Unit (EU)	MOOH area	No. of PHM tested	No. people tested	No. of Mf positive (%, 95% CI)	No. of PHM tested	No. of pools tested	No. of pools positive (%, 95% CI)
**Coastal**	Akmeemana	20	2341	4 (0.2, 0.07–0.4)	3	93	18 (19, 12.6–28.5)
	Ambalangoda	18	1940	1 (0.1, 0.01–0.3)	3	69	7 (10, 5.0–19.5)
	Balapitiya	22	2445	22 (0.9, 0.6–1.4)^a^	3	68	26 (38, 27.6–50.1)^a^
	Bope-poddala	16	2271	1 (0.04, 0.01–0.2)	2	44	2 (4.5, 1.3–15.1)
	Elpitiya	23	2145	0 (0)	4	71	0 (0)
	Galle MC	15	3146	13 (0.4, 0.2–0.7)^a^	2	60	16 (26.7, 17.1–39)^a^
	Gonapinuwala	8	793	1 (0.1, 0.02–0.7)	1	17	0 (0)
	Habaraduwa	19	2339	2 (0.1, 0.02–0.3)	3	39	11 (28, 16.5–43.7)
	Hikkaduwa	34	3795	3 (0.1, 0.03–0.2)	4	71	8 (11, 5.8–20.6)
	Induruwa/Bentota	16	1801	0 (0)	3	45	4 (9, 3.5–20.7)
	Karandeniya	18	2072	0 (0)	2	48	0 (0)
	Total	209	25088	47 (0.2, 0.1–0.2)	30	625	92 (14.7, 12.2–17.7)
**Inland**	Baddegama	25	2719	0 (0)	6	138	0 (0)
	Imaduwa	17	1867	1 (0.1, 0.01–0.3)	4	79	6 (7, 3.5–15.6)
	Neluwa	13	1021	2 (0.2, 0.05–0.7)	3	40	0 (0)
	Niyagama	13	1362	0 (0)	3	62	0 (0)
	Thawalama	13	1026	0 (0)	3	50	0 (0)
	Udugama/Nagoda	23	2061	1 (0.1, 0.01–0.3)	5	85	0 (0)
	Weliwitiya-Divithura	10	955	0 (0)	2	48	0 (0)
** **	Yakkalamulla	17	1966	1 (0.1, 0.01–0.3)	4	81	2 (2, 0.7–8.5)
**Total**		131	12977	5 (0, 0.02–0.09)	30	583	8 (1.4, 0.7–2.7)

^a^ Areas with high *W*. *bancrofti* prevalence in humans with microfilariae and filarial DNA rates in mosquitoes.

### Comparison of filarial DNA rates in mosquitoes and microflaremia rates in humans

Table 4 shows Mf survey results and MX results by MOOH area. Note that all PHM areas were sampled in the Mf survey while MX was performed in only a few PHM areas per MOOH area. Despite this difference, the MX and Mf data both indicated widespread persistence of LF in the coastal EU with much lower rates in the inland EU. The two MOOH areas with the highest Mf rates (Balapitiya and Galle) also had the highest MX signals (% mosquito pools positive for filarial DNA). However, not all areas had consistent results, and three MOOH areas (Ambalangoda, Hikkaduwa, and Habaraduwa) with very high MX positivity had low Mf rates. This may have been due to small samples in the Mf surveys or to differences in the areas sampled, as mentioned above.

Two PHM areas (in Imaduwa and Yakkalamulla MOOHs in the inland EU) also had mosquitoes that were positive for filarial DNA (7% and 2% pools positive, Fig 1) and the same MOOH areas had Mf carriers detected by night blood surveys (Mf rate 0.75%) (Table 4). On the other hand, no positive mosquito pools were detected in other PHMs that were located in MOOH areas in the inland EU where Mf carriers were identified (Table 4). Again, this may be because not all PHMs were sampled for MX, and because persistent filarial infections following MDA can be highly focal.

Table 5 summarizes Mf and MX results for 15 PHM areas in 6 coastal MOOH areas that were tested by both survey methods. These results illustrate the dramatic heterogeneity of persistent *W*. *bancrofti* in Galle district. They also show that MX surveys based on relatively small numbers of mosquito pools are more sensitive for detecting persistent *W*. *bancrofti* infections than Mf testing with sample sizes in the range of 91–267 per PHM area. Only 5 of 15 PHMs had positive signals by Mf testing while 10 of 15 PHMs had positive mosquito pools by qPCR. On the other hand, one PHM with no positive mosquito pool (out of 17 tested) had a positive Mf signal with 1 positive smear out of 101 tested.

**Table 5:** Microfilaremia in humans and filarial DNA rates in mosquitoes by Public Health Midwife area in the coastal evaluation unit.

MOOH area	PHM	No. people tested	No. (%, CI) of Mf positive	No. of mosquito pools tested	Number of Mosquitoes tested	No. (%) of pools positive
Ambalangoda	Patabendumulla	96	0	17	425	6 (35)
	Batapola Central	103	0	31	775	1 (1)
	Diddeliya	101	0	21	525	0
Balapitiya	Wathugedra	113	0	24	600	9 (37)
	Brahmanawatta-N	109	1 (1)	20	487	12 (60)
	Galvehera	130	0	24	600	5 (21)
Galle	Megalle	267	0	32	783	6 (19)
	Kumbhalwella	255	7 (3,1.3–5.6)	28	700	10 (36)
Gonapinuwala	Gonapinuwala	102	1 (1,0.2–5.3)	17	421	0
Bopepoddala	Kapuhempala	151	0	24	586	0
	Ukwattha-East	109	0	20	500	2 (10)
Hikkaduwa	Gammedegoda	112	0	21	525	5 (24)
	Katudampe	119	0	11	275	0
	Wawulagoda	101	1 (1,0.2–5.4)	17	425	3 (18)
	Weragoda	91	0	22	550	0

### Spatial distribution of filarial DNA in fed or gravid *Culex quinquefasciatus*

Fig 3 shows the location of gravid traps that yielded mosquitoes that were tested for filarial DNA by qPCR. Mosquitoes with filarial DNA were widely distributed in coastal areas and also present in a few inland areas. S1 Fig shows a more detailed map for MX results from three PHM areas that were tested in the Balapitiya MOOH area. Filarial DNA was detected in mosquitoes collected in 58% of trapping sites in Balapitiya. These results show that MX is useful for identifying areas with persistent filariasis, and this information could be useful for targeting additional measures to interrupt transmission.

## Discussion

Prior studies have shown persistence of *W*. *bancrofti* in populations following MDA in several areas in Sri Lanka including Galle district, despite the fact that all EUs in the country easily passed TAS performed according to WHO guidelines. This study is the first to demonstrate the value of MX for detecting persistent filarial infection at the level of EUs with large populations. The study was designed to compare MX results for two EUs within one district that were previously shown to have very different rates of persistent infection based on Mf testing and TAS results.

The MX results confirmed that there was much more *W*. *bancrofti* infection in the coastal EU than in the inland EU, and they also showed that transmission was widespread in the coastal EU (population 610,000) and not confined to one or two small hotspots. The results also showed that MX was more sensitive than night blood surveys for detecting persistent *W*. *bancrofti* activity in this study area. We found that MX is feasible for national LF elimination programs. Areas identified as possible hot spots by MX can be followed up with Mf surveys and targeted for intervention with MDA or other options. However, use of MX as a primary surveillance tool requires adequate laboratory facilities, access to supplies, and specially trained personnel required for this method. As a practical matter, the laboratory analysis of mosquitoes is less challenging than the mosquito collection activities, which take a long time and are labor intensive. However, the fact that similar results were obtained by MX in both EUs when the same number of mosquitoes was collected from fewer collection sites is very important, because this would greatly reduce costs and increase feasibility for programs. While it is possible that even fewer mosquito trap sites would have yielded similar results, placement of 75 or 150 traps in 30 randomly selected PMHs for collection of 300 pools of 25 mosquitoes/pool per EU should be feasible for programs interested in using MX as a surveillance method. This result was somewhat surprising, and it is possible that mosquito infection rates in these study areas were unusually homogenous. Additional data will be needed from different areas to establish whether results from this study are generally applicable to areas with *Culex* transmission of *W*. *bancrofti*. We prefer not to speculate regarding implications of this study for post-MDA assessments of areas with filariasis transmission by *Anopheles*, *Mansonia*, or *Aedes* mosquitoes.

Despite these encouraging results, we recognize that the approach we have used for MX is not feasible for all national LF elimination programs. One alternative to consider would be to send mosquitoes to reference centers for qPCR analysis. Another alternative would be to use traditional dissection and staining methods to detect filarial infection and infectivity in mosquitoes [24–26] even though it is clear that dissection with microscopy is less sensitive than qPCR. While we do not have firm break points for *W*. *bancrofti* transmission by *Culex*, reviews have suggested provisional targets of 0.085% for infectivity and 0.65% for mosquito infection by any parasite stage based on dissection with microscopy [23,27].

An additional point to consider is whether results from this study would also apply in areas where filarial parasites are transmitted by *Anopheles* or *Aedes* mosquitoes. Gravid traps work well for trapping large quantities of *Culex* mosquitoes that have fed on human blood, and large numbers of mosquitoes are needed for accurate estimation of parasite DNA rates in mosquitoes [4,27,28]. Human landing catches and indoor sample collections are less effective than gravid traps for collecting mosquitoes for MX. It may be possible to adapt sampling methods used in this study for use with newer mosquito traps that can be used to collect *Anopheles* or *Aedes* vectors [29–33].

MX results obtained in this study were consistent with results obtained by extensive night blood Mf surveys that tested some 38,000 people, which comprised about 3.5% of the population of Galle district. Like MX, Mf testing on this scale is also expensive and labor intensive. In addition, mosquito collection using outdoor traps is less intrusive than collection of finger prick blood samples at night from human subjects. Mf testing showed that 12 of 340 PHM areas tested had Mf rates ≥1%. Since PHM areas are equivalent to sentinel or spot check sites in most countries, EUs with many PHM areas with Mf rates >1% should not have qualified for TAS. The fact that the coastal Galle EU easily passed TAS illustrates the insensitivity of TAS as a post-MDA surveillance tool in Sri Lanka.

Although the WHO has suggested the use of MX to supplement TAS [26,34], it has not provided practical guidelines for systematic sampling of mosquitoes. Results of this study should be helpful for those who are interested in using MX as a surveillance tool at the EU level. One limitation of the mosquito sampling protocol employed in this study is that mosquitoes were only collected from about 20% of the clusters (60 of 340 PHMs) in the coastal and inland EUs. However, some systematic sampling is necessary, because it is not possible to collect random samples of mosquitoes from all areas in an EU. Fortunately, results in Table 3 show that parasite DNA rates were remarkably similar when parasite DNA rates were calculated for 30 different samples of 30 PHMs drawn from the 60 PHMs sampled in this study. This result provides support for the validity of the sampling scheme used in this study. Results in Table 3 also emphasize the importance of using EUs with moderate population sizes that do not mix areas with vastly different risks for persistent *W*. *bancrofti*. Districts are often used as EUs. When data from the coastal and inland EU were combined, the strong signal of persistent *W*. *bancrofti* infection in the coastal area was diluted by negative results from the inland area in a way that could obscure the need for further intervention in the coastal area.

The AFC considered results from this study and other post-MDA surveillance data from Galle district and decided to resume MDA in the coastal areas of the district and simply observe the lower risk inland areas. A repeat MX survey could be used to assess the impact of two additional rounds of MDA that were distributed in September of 2014 and 2015. Time will tell whether MDA alone will solve the problem of persistent *W*. *bancrofti* in coastal Galle or whether anti-mosquito measures or other interventions will be needed in this area.

### Conclusions

The Sri Lanka Anti Filariasis Campaign has succeeded in most formerly endemic areas, and the program has fulfilled many of the criteria required for validation according to WHO guidelines. A prior study compared several different types of tests for detecting persistent *W*. *bancrofti* at the level of Public Health Inspector areas with populations of ~25,000. This study has used MX and Mf testing to document persistence of *W*. *bancrofti* in EUs with populations of 500,000 or more. It is well known that the last mile is often the most difficult for elimination programs. The last 5% is as important as the first 95% when programs are in eradication mode [35]. We have shown that MX can be used as a sensitive method for detecting and mapping areas with persistent *W*. *bancrofti*. This type of information could be very helpful for planning interventions to carry programs across the last mile to the finish line.

## Supporting Information

S1 Fig The map shows molecular xenomonitoring results obtained with mosquito pools collected from gravid traps placed in 3 PHM areas within the Balapitiya MOOH. Waypoints in red indicate trap locations with 1 or more pools positive for filarial DNA, and locations with no positive pools are shown in green. Fifty-eight % of trap locations in Balapitiya yielded one or more mosquito pools that contained filarial DNA.
(TIFF)

## References

[1] WHO (2015) Global programme to eliminate lymphatic filariasis: progress report, 2014. Wkly Epidemiol Rec 90: 489–504. 26387149

[2] Ramaiah KD, Ottesen EA (2014) Progress and impact of 13 years of the global programme to eliminate lymphatic filariasis on reducing the burden of filarial disease. PLoS Negl Trop Dis 8: e3319 10.1371/journal.pntd.0003319 25412180 PMC4239120

[3] Hooper PJ, Chu BK, Mikhailov A, Ottesen EA, Bradley M (2014) Assessing progress in reducing the at-risk population after 13 years of the global programme to eliminate lymphatic filariasis. PLoS Negl Trop Dis 8: e3333 10.1371/journal.pntd.0003333 25411843 PMC4239000

[4] Rao RU, Nagodavithana KC, Samarasekera SD, Wijegunawardana AD, Premakumara WD, et al (2014) A comprehensive assessment of lymphatic filariasis in Sri Lanka six years after cessation of mass drug administration. PLoS Negl Trop Dis 8: e3281 10.1371/journal.pntd.0003281 25393404 PMC4230885

[5] Ramzy RM, El Setouhy M, Helmy H, Ahmed ES, Abd Elaziz KM, et al (2006) Effect of yearly mass drug administration with diethylcarbamazine and albendazole on bancroftian filariasis in Egypt: a comprehensive assessment. Lancet 367: 992–999. 16564361 10.1016/S0140-6736(06)68426-2

[6] Farid HA, Morsy ZS, Helmy H, Ramzy RM, El Setouhy M, et al (2007) A critical appraisal of molecular xenomonitoring as a tool for assessing progress toward elimination of Lymphatic Filariasis. Am J Trop Med Hyg 77: 593–600. 17978055 PMC2196407

[7] Weil GJ, Kastens W, Susapu M, Laney SJ, Williams SA, et al (2008) The impact of repeated rounds of mass drug administration with diethylcarbamazine plus albendazole on bancroftian filariasis in Papua New Guinea. PLoS Negl Trop Dis 2: e344 10.1371/journal.pntd.0000344 19065257 PMC2586652

[8] Bockarie MJ (2007) Molecular xenomonitoring of lymphatic filariasis. Am J Trop Med Hyg 77: 591–592. 17978054

[9] WHO Neglected tropical diseases. PCT data bank. Accessed on April 20, 2016.: http://www.who.int/neglected_diseases/preventive_chemotherapy/lf/en/.

[10] Abdulcader MH (1965) An outline of the problem and control of filariasis in Ceylon. Ceylon Med J 10: 64–66. 5831957

[11] Lambrecht FL (1974) Entomological aspects of filariasis control in Sri Lanka. Bull World Health Organ 51: 133–143. 4619057 PMC2366225

[12] Schweinfurth U (1983) Filarial diseases in Ceylon: a geographic and historical analysis. Ecol Dis 2: 309–319. 6152421

[13] Dissanaike AS (1991) Filariasis in Ceylon then (1961) and in Sri Lanka now (1990–30 years on). Ann Trop Med Parasitol 85: 123–129. 1888209 10.1080/00034983.1991.11812538

[14] Dissanayake S, Jayasekera N (1989) Bancroftian Filariasis in Sri Lanka: An overview of current knowledge. J Natn Sci Coun Sri Lanka 17: 141–160.

[15] Henderson RH (2011) Introduction Xenomonitoring: Sampling issues for Lymphatic filariasis. Xenomonitoring for Lymphatic Filariasis. Meeting report Atlanta, GA. 1–13 p.

[16] Irish SR, Moore SJ, Derua YA, Bruce J, Cameron MM (2013) Evaluation of gravid traps for the collection of *Culex quinquefasciatus*, a vector of lymphatic filariasis in Tanzania. Trans R Soc Trop Med Hyg 107: 15–22. 10.1093/trstmh/trs001 23222942

[17] Williams SA, Laney SJ, Bierwert LA, Saunders LJ, Boakye DA, et al (2002) Development and standardization of a rapid, PCR-based method for the detection of *Wuchereria bancrofti* in mosquitoes, for xenomonitoring the human prevalence of bancroftian filariasis. Ann Trop Med Parasitol 96 Suppl 2: S41–46. 12625916 10.1179/000349802125002356

[18] Rao RU, Atkinson LJ, Ramzy RM, Helmy H, Farid HA, et al (2006) A real-time PCR-based assay for detection of *Wuchereria bancrofti* DNA in blood and mosquitoes. Am J Trop Med Hyg 74: 826–832. 16687688 PMC2196401

[19] Weil GJ, Ramzy RM (2007) Diagnostic tools for filariasis elimination programs. Trends Parasitol 23: 78–82. 17174604 10.1016/j.pt.2006.12.001

[20] Lwanga SK, Lemeshow S (1991) Sample size determination in health studies: A practical manual World Health Organization Geneva: 1–77.

[21] Katholi CR, Toe L, Merriweather A, Unnasch TR (1995) Determining the prevalence of *Onchocerca volvulus* infection in vector populations by polymerase chain reaction screening of pools of black flies. J Infect Dis 172: 1414–1417. 7594692 10.1093/infdis/172.5.1414

[22] Katholi CR, Unnasch TR (2006) Important experimental parameters for determining infection rates in arthropod vectors using pool screening approaches. Am J Trop Med Hyg 74: 779–785. 16687680

[23] WHO (2009) The role of polymerase chain reaction techniques for assessing lymphatic filariasis transmission. WHO/HTM/NTD/PCT/20091: 1–59.

[24] Suzuki T, Seregeg IG (1979) A mass dissection technique for determining infectivity rate of filariasis vectors. Jpn J Exp Med 49: 117–121. 384057

[25] Nelson GS (1958) Staining of filarial larvae in insects before dissection. Bull World Health Organ 19: 204 13585072 PMC2537700

[26] WHO (2013) Lymphatic Filariasis: Practical Entomology. A Handbook for National Elimination Programmes. WHO/HTM/NTD/PCT/201310: 1–92.

[27] Pedersen EM, Stolk WA, Laney SJ, Michael E (2009) The role of monitoring mosquito infection in the Global Programme to Eliminate Lymphatic Filariasis. Trends Parasitol 25: 319–327. 10.1016/j.pt.2009.03.013 19559649

[28] Irish SR, Stevens WM, Derua YA, Walker T, Cameron MM (2015) Comparison of Methods for Xenomonitoring in Vectors of Lymphatic Filariasis in Northeastern Tanzania. Am J Trop Med Hyg 93: 983–989. 10.4269/ajtmh.15-0234 26350454 PMC4703286

[29] Schmaedick MA, Ball TS, Burkot TR, Gurr NE (2008) Evaluation of three traps for sampling *Aedes polynesiensis* and other mosquito species in American Samoa. J Am Mosq Control Assoc 24: 319–322. 18666543 10.2987/5652.1

[30] Schmaedick MA, Koppel AL, Pilotte N, Torres M, Williams SA, et al (2014) Molecular xenomonitoring using mosquitoes to map lymphatic filariasis after mass drug administration in American Samoa. PLoS Negl Trop Dis 8: e3087 10.1371/journal.pntd.0003087 25122037 PMC4133231

[31] Govella NJ, Chaki PP, Geissbuhler Y, Kannady K, Okumu F, et al (2009) A new tent trap for sampling exophagic and endophagic members of the *Anopheles gambiae* complex. Malar J 8: 157 10.1186/1475-2875-8-157 19602253 PMC2720981

[32] Okumu FO, Madumla EP, John AN, Lwetoijera DW, Sumaye RD (2010) Attracting, trapping and killing disease-transmitting mosquitoes using odor-baited stations—The Ifakara Odor-Baited Stations. Parasit Vectors 3: 12 10.1186/1756-3305-3-12 20193085 PMC2838860

[33] Hapairai LK, Plichart C, Naseri T, Silva U, Tesimale L, et al (2015) Evaluation of traps and lures for mosquito vectors and xenomonitoring of *Wuchereria bancrofti* infection in a high prevalence Samoan Village. Parasit Vectors 8: 287 10.1186/s13071-015-0886-2 26016830 PMC4449966

[34] WHO (2012) Transmission assessment surveys in the Global Programme to Eliminate Lymphatic Filariasis: WHO position statement. Wkly Epidemiol Rec Geneva: World Health Organization 30: 478–482. 23213667

[35] Cockburn TA (1961) Eradication of infectious diseases. Science 133: 1050–1058. 13694225 10.1126/science.133.3458.1050

