# Supplementary figures and images for: Programmatic Use of Molecular Xenomonitoring at the Level of Evaluation Units to Assess Persistence of Lymphatic Filariasis in Sri Lanka
Source: PLoS Negl Trop Dis. 2016 May 19;10(5):e0004722. doi: 10.1371/journal.pntd.0004722 (PMC4873130; doi:10.1371/journal.pntd.0004722)

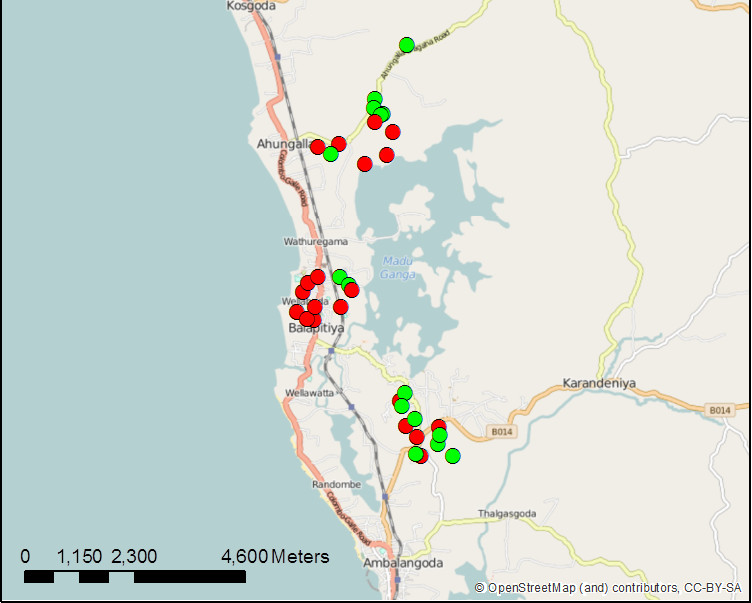

Supplement: S1 Fig — Waypoints in red indicate trap locations with 1 or more pools positive for filarial DNA, and locations with no positive pools are shown in green. Fifty-eight % of trap locations in Balapitiya yielded one or more mosquito pools that contained filarial DNA. (TIFF) [file pntd.0004722.s001.tiff]
